# The mechanistic effects of acupuncture in rodent neurodegenerative disease models: a literature review

**DOI:** 10.3389/fnins.2024.1323555

**Published:** 2024-03-04

**Authors:** Boxuan Li, Shizhe Deng, Hailun Jiang, Weiming Zhu, Bifang Zhuo, Yuzheng Du, Zhihong Meng

**Affiliations:** ^1^National Clinical Research Center for Chinese Medicine Acupuncture and Moxibustion, Tianjin, China; ^2^First Teaching Hospital of Tianjin University of Traditional Chinese Medicine, Tianjin, China

**Keywords:** acupuncture, neural circuit, movement disorder, cognitive impairment, neurodegenerative disease

## Abstract

Neurodegenerative diseases refer to a battery of medical conditions that affect the survival and function of neurons in the brain, which are mainly presented with progressive loss of cognitive and/or motor function. Acupuncture showed benign effects in improving neurological deficits, especially on movement and cognitive function impairment. Here, we reviewed the therapeutic mechanisms of acupuncture at the neural circuit level in movement and cognition disorders, summarizing the influence of acupuncture in the dopaminergic system, glutamatergic system, γ-amino butyric acid-ergic (GABAergic) system, serotonergic system, cholinergic system, and glial cells at the circuit and synaptic levels. These findings can provide targets for clinical treatment and perspectives for further studies.

## 1 Introduction

Neurodegenerative diseases are characterized by progressive brain dysfunction and overlapping clinical syndromes induced by damage to neurons and synapses (Gan et al., 2018). Movement disorder and cognitive deficits are common complaints in neurodegenerative disorders, such as Parkinson’s disease (PD) and Alzheimer’s disease (AD) (Shi et al., 2020). Given the considerable prevalence of neurodegenerative diseases, more than 52 million people were affected by AD and dementia globally in 2019, including 3.9 million individuals with PD, with a 1.61- and 1.56-fold increase from 1990, respectively (Cieza et al., 2021). With the booming population and prolonged life expectancy, it is estimated that there will be 42.5 million people with AD in China by 2050, and the number will be 152 million worldwide (Nichols et al., 2022). The impairment of motor and cognitive function frustrates normal daily life and lowers the quality of life (Tahami Monfared et al., 2022; Leite Silva et al., 2023). The societal expenditure on dementia has increased by 161% from 2010 to 2015 in the USA (Dauphinot et al., 2022), and economic studies forecast 332 billion RMB costs of AD in 2050 in China (Clay et al., 2019). For PD, the individual healthcare economic burden on physiotherapy ranges from €2056 to €2,586 annually (Ypinga et al., 2018), and the projected PD expense will be at least $79 billion in the USA by 2037 (Yang et al., 2020). Regarding the growing demographic trends, the burden of people with neurodegenerative diseases will continue to grow.

As the aggregation and spreading of abnormal proteins are shared pathogenetic mechanisms in the neural degeneration process, similar metabolic connectivity changes have been reported at the brain network level (Boccalini et al., 2022). Well-organized neural circuits via precise complex connectivity between neurons have been recognized as the foundation of specific neural functions. Classical neural circuits are neural networks comprising axons, dendritic terminals, and glial cells, which perform functions using specific excitation or inhibition signal flow pathways (Luo, 2021). For motor control, the basal ganglia neural circuit accounts for subtle movement modulation, whose dysfunction following dopamine (DA) loss plays a critical role in PD (Mcgregor and Nelson, 2019). In cognitive function, the cholinergic circuit from the medial septal and vertical limb of the diagonal band (VDB) to the hippocampus with cholinergic neuron damage contributes to learning deficits and memory impairment in AD model rats (Liu et al., 2022). Synaptic plasticity provides a rehabilitation foundation for motor learning and cognitive function improvement at the synapse level (Chu, 2020). Sufficient evidence has proven adaptive alterations in synapses in neurodegenerative disorders, such as synaptic changes in the striatum and the subthalamic nucleus in PD (Shen et al., 2022) and long-term potentiation (LTP) alterations in the hippocampal neural circuit in cognition modulation (Cuestas Torres and Cardenas, 2020).

Acupuncture provides an adjunctive approach to the rehabilitation of neurological disorders (Shi, 2021; Lu et al., 2022; Zhang Z. et al., 2023). Clinical studies have validated the neurological functional improvement effect of acupuncture in cognitive impairment (Yang et al., 2019) and movement disorders. For the patients with vascular cognitive impairment, acupuncture showed indeed effects in improving cognitive symptom and preventing the process of dementia (Lei et al., 2016; Jang et al., 2020a). As for PD patients with gait disturbance, acupuncture benefits the hypometric gait and functional brain activity, providing a practical approach for PD. Acupuncture plays a role in improving anatomical and functional changes in the brain network via multiple signaling pathways, on both the neural circuit and synapse level (Lin et al., 2022; Liu and Zhu, 2023). Recently, the therapeutic mechanisms of acupuncture at the neural circuit level have been frequently reported in neurological deficits, especially in the dopaminergic system, glutamatergic system, γ-amino butyric acid-ergic (GABAergic) system (Fan et al., 2022), serotonergic system, and cholinergic system (Li L. et al., 2021). In addition, glial cells such as astrocytes, microglia, and oligodendrocytes (OLs) contribute to neural plasticity by facilitating neural circuit rehabilitation (Allen and Lyons, 2018), of which the mechanism of acupuncture interference has been demonstrated (Chang et al., 2022). At the synapse level, mechanisms of acupuncture on synaptic plasticity have been revealed in modulating ion channels, functional proteins, and neurotransmitters (Xiao et al., 2018).

Although sufficient evidence has proven the mechanistic pathways of acupuncture in neurodegenerative diseases, limited reviews focus on motor disorders and cognitive impairment at the circuit and synaptic levels. In this review, we summarized the neural protective effects and therapeutic mechanisms of acupuncture on movement and cognitive disorders in neurodegenerative diseases.

## 2 Influence of different neuronal and glial systems on movement and cognitive disorders

### 2.1 Neuronal systems

#### 2.1.1 Dopaminergic system

Dopaminergic neurons are mainly distributed in the dense region of the substantia nigra (SN) of the midbrain, generating neurofibers that project to the telencephalon, diencephalon, brainstem, and spinal cord, and form dopaminergic neuronal pathways, including the nigra-striatum network, mesocortical pathway, mesolimbic pathway, and tuberoinfundibular system (Morita and Kawaguchi, 2019).

The basal ganglia modulates the control and initiation of movement via two neural circuits projecting from the striatum (Rui et al., 2013): one is the direct circuit comprised of dopaminergic neurons with the D1 receptor, and the other is the indirect pathway comprised of dopaminergic neurons with the D2 receptor (Yan, 2021). The dynamic balance of the basal ganglia-thalamocortical circuits is crucial for normal activity.

In neurodegenerative diseases, the integrity of dopaminergic neural circuits and the activation of the dopamine receptor account for normal movement performance (Klein et al., 2019). One hypothesis is the release of striatal dopamine regulates movement control, which accounts for movement disorders such as Parkinson’s (Geibl et al., 2019); another is the distribution of dopamine receptors plays a role in the formation of LTP or long-term depression (LTD), which contributes to the cognitive impairment (Jay, 2003).

#### 2.1.2 Glutamatergic system

The glutamatergic system contains glutamatergic neurons, glutamate transporters, glutamate receptors, and glutamate neurotransmitters. Glutamatergic (Hussan et al., 2022) neurons form a complex neural network primarily comprising the cortex-to-striatum pathway, the hippocampus-to-septum pathway, the visual and auditory systems, the olfactory system, and the cerebellum (Broman et al., 2004; Hussan et al., 2022).

**Figure 1:**
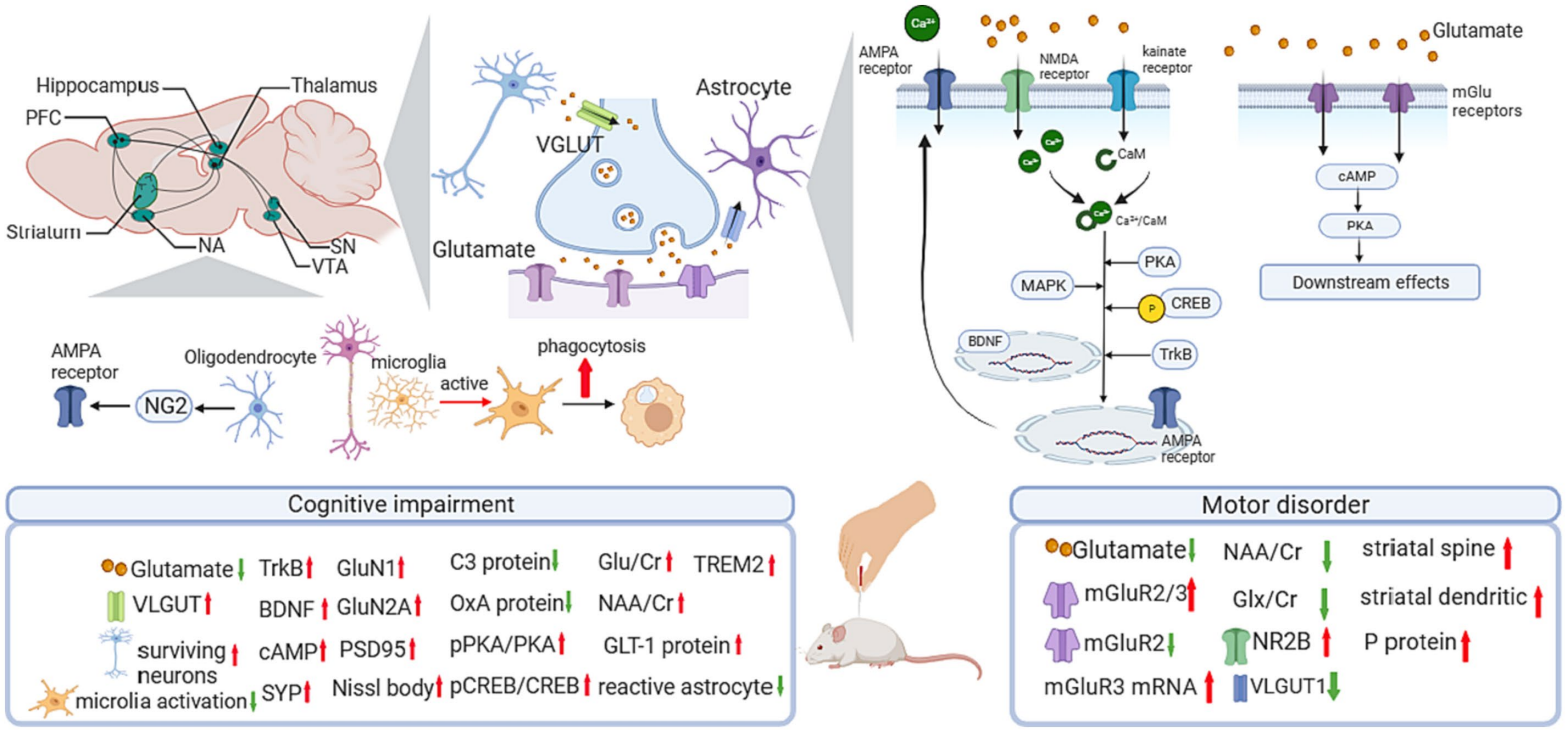
Influence of acupuncture on the glutamatergic system. AMPA, α-amino-3-hydroxy-5-methyl-4-isoxazole propionic acid; BDNF, brain-derived neurotrophic factor; C3, compliment component 3; CaM, calmodulin; cAMP, cyclic adenosine monophosphate; Cr, creatine; CREB, cAMP response element-binding protein; GLT-1, glutamate transporter 1; Glu, glutamine; Glx, glutamate + glutamine; mGluR, metabotropic glutamate receptor; NA, nucleus accumbens; NAA, N-acetylaspartate; NG2, nerve-glia antigen 2; NMDA, N-methyl-D-aspartate; NR2B, receptor subunit 2B; OxA, Orexin A; PFC, prefrontal cortex; PKA, cAMP response element-binding protein; pPKA, cAMP response element-binding protein; pPKA, phosphorylated PKA; PSD95, postsynaptic density protein 95; SN, substantia nigra; TrkB, Tyrosine Kinase receptor B; VTA, ventral tegmental area; VGLUT, vesicular Glu transporter.

In neurodegenerative disorders, two vital processes related to the glutamatergic system have been reported, one is the excessive accumulation of glutamic acid (Glu) causing neuronal swelling and membrane integrity destruction, hence leading to movement disorders (Hirschberg et al., 2022) and cognitive impairment (Conway, 2020); another is the imbalance between synaptic and extrasynaptic glutamatergic receptors (Babaei, 2021), including the N-methyl-D-aspartate (NMDA) receptors that induce excitatory postsynaptic potential (EPSP), and the aminomethylphosphonic acid (AMPA) receptors that facilitate the formation of late long-term potentiation (L-LTP) (Bonansco et al., 2023; Figure 1).

#### 2.1.3 GABAergic system

The GABAergic system comprises γ-amino butyric acid (GABA), GABA transporters, and GABA receptors. As the cardinal inhibitory system in the central neural system (CNS), the GABAergic system contributes to the formation of synaptic plasticity, participating in the modulation of learning and memory (Zhang et al., 2021). Ligand-gated ion channel GABA type A receptors are the major mediator of fast inhibition in the CNS, regulating the balance of excitatory and inhibitory (E/I) synapses alone with Glu receptors (Chapman et al., 2022).

Growing evidence has proven the relationship between dysfunction of the GABAergic system and neural disorders, especially in the over-activity of hippocampal neurons in cognitive impairment, and the enhanced excitability in the primary motor cortex in movement disorder.

#### 2.1.4 Serotonergic system

**Figure 2:**
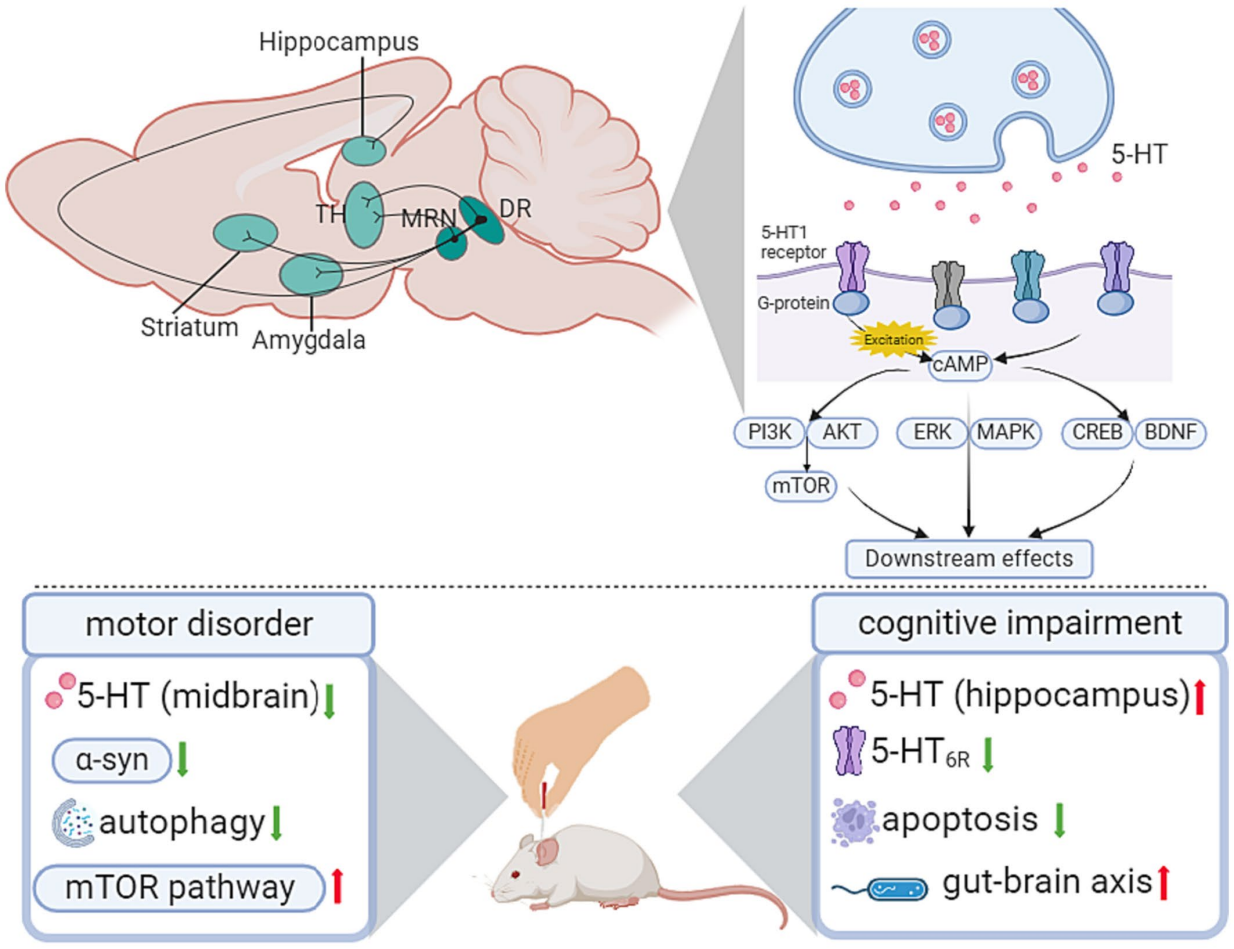
Influence of acupuncture on the serotonergic system. AKT, protein kinase B; cAMP, cyclic adenosine monophosphate; BDNF, brain-derived neurotrophic factor; CREB, cAMP response element-binding protein; DR, dorsal raphe; ERK, extracellular signal-regulated kinase; LDT, laterodorsal tegmental nucleus; MAPK, mitogen-activated protein kinase; MRN, median raphe nucleus; mTOR, mammalian target of rapamycin; PI3K, phosphatidylinositol 3-kinase; TH, thalamus.

Serotonin is also known as 5-hydroxytryptamine (5-HT), which is a neuromodulatory neurotransmitter secreted by serotonergic neurons. Serotonergic neurons are mainly distributed in brainstem raphe nuclei, from which projections are sent to wide areas of the brain (Paquelet et al., 2022; Figure 2). A battery of canonical signaling pathways is involved in the 5-HT-mediated system, including the phosphatidylinositol 3-kinase/protein kinase B/ mammalian target of rapamycin (PI3K-Akt/mTOR) signaling pathway, extracellular signal-regulated kinase/mitogen-activated protein kinase (ERK/MAPK) signaling pathway (Albert and Vahid-Ansari, 2019), and cAMP response element-binding protein/cAMP response element-binding protein (PKA/CREB) pathway (Yue et al., 2022; Lin et al., 2023).

The dysfunction of 5-HT and its receptors has been validated to be associated with different phases of neurodegenerative disease. In movement disorder-related research, the inactivation of 5-HT neurons of the dorsal raphe nucleus (DRN) accounts for the impaired behavioral flexibility in the DRN-cerebral cortex projection (Hale and Lowry, 2011). As for cognitive impairment, the expression level of 5-HT receptors in the hippocampus contributes to spatial memory deficits (Crispino et al., 2020).

#### 2.1.5 Cholinergic system

The cholinergic system is known to be associated with memory and learning ability. In the CNS, cholinergic neurons are mainly distributed in the basal forebrain, brainstem pedunculopontine and lateral dorsal tegmental nuclei, striatum, and cortex. The basal forebrain cholinergic system comprises 4 distinct nuclei projecting to specific forebrain targets, including the medial septal (Ch1), VDB (Ch2), horizontal limb of the diagonal band (Ch3), and basalis of Meynert (Ch4)(Mesulam, 2013). Among them, Ch4 has been reported to be correlated with cognitive impairment progression in patients with PD (Zhang P. et al., 2023). The projection from the basal forebrain cholinergic system to the hippocampus, amygdala, and cortex plays a crucial role in cognitive function and motor modulation.

Previous studies have illustrated the relationship between cholinergic neuron degeneration and cognitive dysfunction in dementia patients (Berry and Harrison, 2023), thus proposing the classical “cholinergic hypothesis”(Hampel et al., 2018). Recently, studies reported enlightening findings on the cholinergic system in AD treatment. Pharmaceutical treatments, such as acetylcholinesterase inhibitors, have modest effects in different phases of dementia (Joe and Ringman, 2019).

In the nucleus basalis-cortical projection, the tauopathy in the cholinergic system leads to the degeneration of the cholinergic axons, accounting for cognitive impairment in AD (Mesulam, 2013). As for PD, the activation of striatal cholinergic system accelerates the suppression of the direct pathway and the overexcitation of the indirect pathway, which aggravates the symptom of PD (Liu, 2020).

### 2.2 Glial cells

In the CNS, neuroglial cells consist of astrocytes, microglia, and oligodendrocytes and facilitate neural functions at the neural circuit, cellular, and molecular levels (Oliveira and Araque, 2022). Neuroglia performs function through alternating neuroprotective and neurotoxic effects, which are essential to the homeostasis of the CNS (Sancho et al., 2021).

#### 2.2.1 Astrocytes

Astrocytes are the major type of glial cells in the CNS and facilitate brain activity, and mediate neuronal activity by controlling the extracellular microenvironment of ions and neurotransmitters at the synaptic level. The activity of Ca^2+^ in the astrocyte network has been proven to be influenced by neurotransmitters in different neural circuits, including the prefrontal cortex with the GABAergic neural pathway, the cortex and hippocampus with the cholinergic circuit, and the cortex and inferior colliculus with the glutamatergic pathway (Oliveira and Araque, 2022).

Research has demonstrated that the enhanced intracellular Ca^2+^ in hippocampal astrocytes alters the memory formation process. In the medial prefrontal cortex, activated astrocyte Ca^2+^ increased cortical activity and improved cognition (Mederos et al., 2021). In addition, astrocytes interfere with neuronal oscillations by modulating ion content and ion buffering and regulating neuronal firing patterns and neuronal synchronization in several neural networks (Brockett et al., 2018). In prefrontal cortex astrocytes, the deletion of GABAB receptors altered low-gamma oscillations and firing properties of cortical neurons, which resulted in poor working memory (Mederos et al., 2021).

#### 2.2.2 Microglia

Microglia are commonly recognized as the brain’s resident immune cells, which are crucial for inflammatory/repair responses in the CNS. Studies have investigated the signaling pathways of microglia-synapse interactions and uncovered the synaptic plasticity regulatory mechanisms of microglia (Yu et al., 2023). The microglia-dependent synapse elimination is a vital phase in the development of neural circuits (Hansen et al., 2018). On the one hand, microglia prompt the formation of synapses by secreting cytokines (Andoh and Koyama, 2021); on the other hand, microglia facilitate synapse elimination via phagocytosis (Hansen et al., 2018).

In PD model rats, activated microglia appeared in the substantia nigra pars reticulata (SNr), and microglia phagocytosed glutamatergic synapses of the STN neurons (Aono et al., 2017). Through phagocytosis, microglia might alleviate PD symptoms by eliminating hyperactive synapses (Choudhury et al., 2021). In an AD model, pathological changes in microglia were observed before tau aggregation pathology (Leng and Edison, 2021).

#### 2.2.3 Oligodendrocytes

Oligodendrocytes are derived from oligodendrocyte progenitor cells (OPCs) (Shengjiao et al., 2022), and act as receptors of GABA and Glu neurotransmitters and facilitate neural plasticity by promoting the function of synaptic receptors. Recent studies have reported that the formation of learning-related neuron circuits is associated with the OPC subgroups. In brain-injured mice that received motor learning stimulation, the formation of Oligodendrocyte Transcription Factor 2 (Olig2)-negative OPCs is related to the establishment of neural circuits in the dentate gyrus (DG) and CA1 regions (Fang et al., 2023).

## 3 Neural circuit mechanism of acupuncture in movement disorders via different neuronal and glial systems

### 3.1 The impact of acupuncture on the dopaminergic system

**Figure 3:**
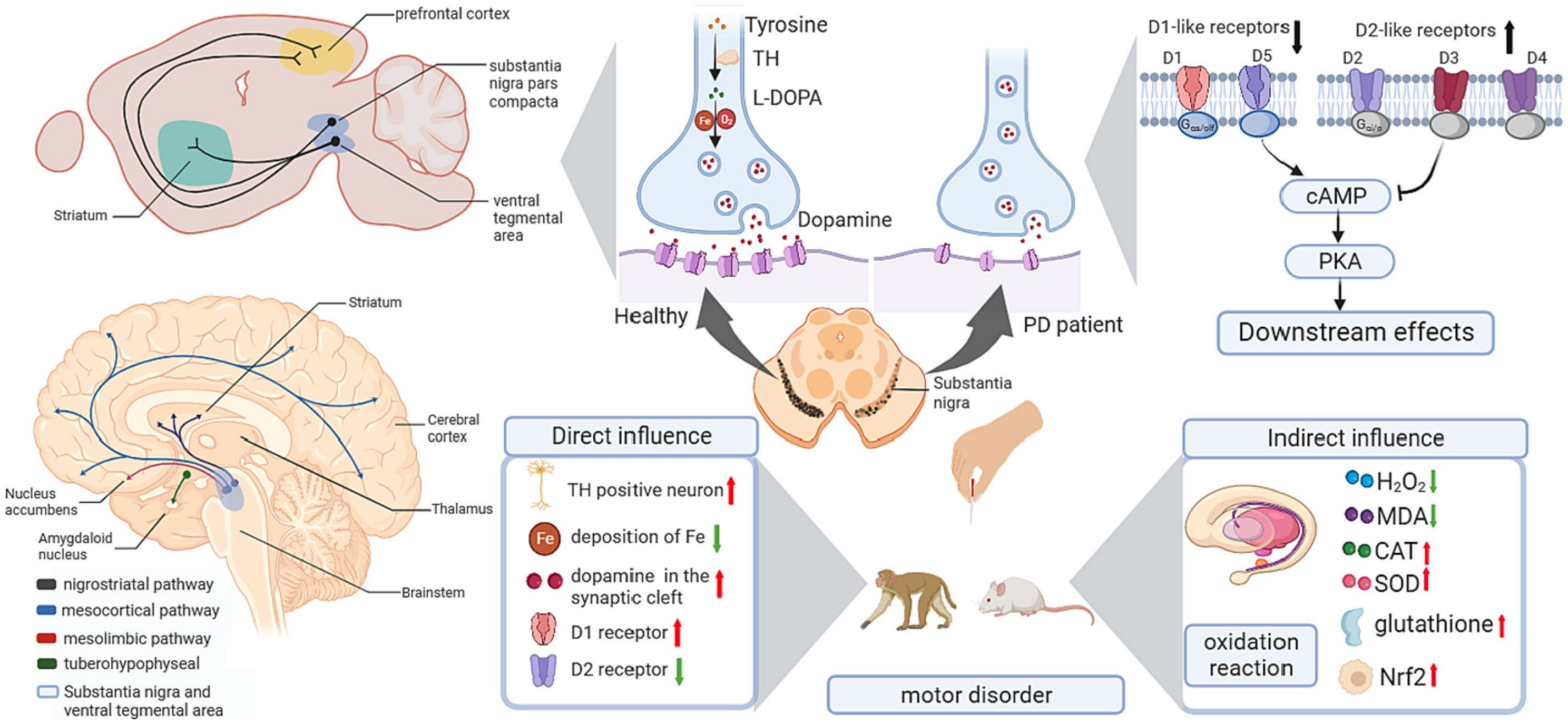
Influence of acupuncture on the dopaminergic system. cAMP, cyclic adenosine monophosphate; CAT, catalase; L-DOPA, levodopa; MDA, malondialdehyde; Nrf2, nuclear factor erythroid 2-related factor 2; PD, Parkinson’s disease; PKA, cAMP response element-binding protein; SOD, superoxide dismutase; TH, Tyrosine hydroxylase.

Acupuncture indicated benign effects in improving motor performance and behavioral disorders, and showed protective effects dopaminergic signaling pathways, including regulating the expression of Tyrosine hydroxylase (TH), DA, and DA receptors targeting the signaling pathways relating to oxidative stress activity, and modulating the expression level of neurotrophic factors (Table 1; Figure 3).

**Table 1:** The effect and mechanisms of acupuncture on movement in neurodegenerative diseases.

Author	Animal model/participant	Acupoint	Acupuncture type	Acupuncture session	Behavioral test	Outcomes
Dopaminergic system						
Pak et al. (2019)	MPTP mice model of PD	GV20, GV14	EA, 2 Hz, 1 mA, 20 min/day	once a day, 10 days	Rotarod test: latency to fall↑	Striatum: mean IOD↑; GFRα-1↓, GFRα-1/TH-positive cell↑; substantia: TH-positive cell↑; cerebrum mBDNF, GDNF↑; ipsilateral (lesioned) substantia nigra and striatum: dopaminergic neuronal loss↓; pCREB↑; pCREB/TH double-positive cell↑; pAkt/TH-positive cell↑; pCREB/pAkt double-positive cell↑; Pitx3/TH double-positive cell↑
Zhang et al. (2018)	MPTP rhesus monkeys model of PD	ST36, LI4	EA, 100 Hz, 4–5 mA, 30 min/day	3 times/week for 12 weeks	Movement speed↑, home cage activity↑, fine motor performance time↓	BOLD-responses: MPTP vs. normal: Cingulate, GPe; MPTP + EA vs. normal: GPi; MPTP + EA vs. MPTP: Cingulate, GPe, GPi; phMRI activation changes after EA: Primary Motor Cortex activation↓
Lin et al. (2017)	MPTP rat model of PD	GB34, LR3	EA, 50 Hz, 1 mA, 20 min/day	9 days	Latency to fall in the rotarod test↑; distance and velocity of behavior and locomotor activity↓	SN: tyrosine hydroxylase-positive neuronal loss↓; striatum: dopaminergic terminal degeneration↓; ipsilateral side of SN: mBDNF↑; tyrosine hydroxylase↑; Bcl-2 expression↑; Akt phosphorylation↑
Lv et al. (2015)	MPTP rat model of PD	ST36, SP6	EA, 100 Hz, 1.4 mA, 30 min/day	once a day, 12 days	OFT: vertical total distance↑, horizontal total distance^#^, rotarod test^#^	Striatum: CAT↑, MDA↓, SOD↑, DNP-modified protein↓, TNF-α↓, IL-6↓, IL-1β↓, GCLM^#^; midbrain and striatum: microglial activation↓, astrogliosis↓; striatum: TH-positive neuron↑, TH protein↑, DAT↑, VMAT2↑; SNpc: TH-positive neuron↑^#^, TH protein↑^#^; midbrain and striatum: hPAP↑, NQO1↑, Nrf2↑, HO-1↑, GCLM^#^
Kim et al. (2014)	6-OHDA induced mice model of PD	GB34	MA	Instant acupuncture,10 days	Rotation test; cylinder wall touches↓; dyskinesia↓; abnormal involuntary movement score↓	Striatum: FosB activation↓; SN: GABA level normalization; Glu↓
Rui et al. (2013)	6-OHDA induced rat model of PD	GB34, SP6, ST36	EA, 0/2/100 Hz, 1–3 mA, 30 min/day	6 times/week for 2 weeks	Rotarod test: rotation number↓; motor disorder symptoms↓	SN: TH-positive neuron number↑, TH-positive dendritic fiber network↑, DA level↑^#^; striatum: D1R mRNA↑, D1R protein↑, D2R mRNA↓, D2R protein↓
Kim et al. (2011)	MPTP mice model of PD	GB34	MA	instant acupuncture, 12 days	Rotarod test: overall rod performance score↑	SN: TH-positive cell↑; SNpc: Nissl-positive cell↑; striatum: dopaminergic fibers↑; striatum: HVA↑, DOPAC/DA↑, HVA/DA↑, DOPAC+HVA/DA↑, Dopamine efflux↑; DARPP-32 phosphorylation at Thr34↓, FosB↓
Glutamatergic system						
Li L. et al. (2021)	6-OHDA induced rat model of PD	GV14, GV20	EA, 100 Hz with 1/2/3 mA, 10 min/day	6 days/week, 4 weeks	Accelerod performance↑, FP locomotion↑	Striatal LTP↑; NR2B↑ in striatum; striatal spine density↑; striatal dendritic↑; striatal glutamate concentration↓; VGLUT-1 in the striatum↓
Li Y. et al. (2020)	6-OHDA induced rat model of PD	GV14, GV20	EA, 100 Hz, 3 mA, 30 min/day	6 days/week, 4 weeks	OFT: P move time↑, FP distance↑, rotarod test: latency to fall↑	NAA/Cr ratios, Glx/Cr ratio in the striatum↓
Wang et al. (2018)	6-OHDA induced rat model of PD	GV14, GV20	EA, 100 Hz, 3 mA, 10 min/day	6 days/week, 4 weeks	Rotation test: intensity of APO-induced rotation↓; rotarod test: latency to fall↓	Lesioned striatum: degeneration of TH-positive profiles^#^; lesioned SNpc: TH-positive neurons^#^; lesioned STN: HSV-GFP-positive neurons^#^, VGLUT-1 levels↑
Jia et al. (2017)	6-OHDA induced rat model of PD	GV14, GV20	EA, 0/100 Hz, 3 mA, 30 min/day	6 days/week, 2/4 weeks	Rotation test: rotation numbers↓; rotarod test: latency↑	Lesioned side of SNpc: TH-positive neuron loss^#^; striatum: TH-positive fibers^#^, mGluR3 mRNA↑, mGluR2↓ mRNA, GluR2/3 proteins↑, extracellular glutamate↓; ventral midbrain: TH expression level^#^; striatum: DA content^#^
Jia et al. (2009)	MFB transection induced rat model of PD	GV14, GV20	EA, 100 Hz, 3 mA, 10 min/day	6 days/week, 4 weeks	Rotation test: net number of rotations↓	SN: TH-positive neurons↑, P content↑; globus pallidus: ENK content^#^; ventral midbrain: GAD67 mRNA↓
GABAergic system						
Jia et al. (2010)	MFB transection induced rat model of PD	GV20, GV14	EA, 0/2/100 Hz, 1 mA, 30 min/day	6 days/week, 2/4 weeks	Rota-Rod test: treadmill occupancy time↑	Ipsilateral striatum: DA level^#^; ventral midbrain: GABA content↓; GP: GABA content^#^
Du et al. (2011)	6-OHDA-induced mice model of PD	GV20, GV14	EA, 100 Hz, 1–3 mA, 30 min/day	7 days	Rotation test: rotation numbers↓	Cortex: GABA content of lesioned side/unlesioned side↓; striatum: GABA content of lesioned side/unlesioned side↑, cerebralleum: GABA content of lesioned side/unlesioned side↑; midbrain: GABA content of lesioned side/unlesioned side^#^
Serotonergic system						
Ning et al. (2023)	6-OHDA-induced mice model of PD	GV20, GV29, LI4, LR3, GV4	MA, 20 min/day	once a day, 28 days	OFT: horizontal activity distance↑, vertical activities number↑; Forced swimming test: immobility time of forced swimming↓; Sucrose preference test: sugar water preference rate↑	Midbrain: DA↑, 5-HT↑; striatum: α-syn, Beclin-1↓, p62↑, mTOR↑, number of autophagosomes↓, p-p70s6k↑, synapsin I↑, PSD95↑; prefrontal cortical neurons: number of autophagosomes↓
Cholinergic system						
Sun et al. (2012)	MFB transection induced rat model of PD	GV14, GV20	EA, 100 Hz, 3 mA, 30 min/day	6 days/week, 4 weeks	Rotation test: net number of rotations↓; rotarod test: treadmill occupancy time↑	SNpc: TH-positive neurons↑; striatum: DA content^#^; ACh↓, Glu levels↓, GABA^#^
Glial cells						
Jang et al. (2020b)	MPTP rat model of PD	GB34, ST36	MA	30 s, instant acupuncture	Motor function tests: rearing test: number of rears↑; akinesia test: latency time↓; rotarod test: latency time↑; OFT: platform crossing number↑	ST: TH optical density↑, TH expression↑, GFAP↓, NF-κB↓, TNF-α↓, Iba-1↓, Bax↓, Bcl-2↑ SN: TH-positive neurons↑, TH expression level↑, GFAP↓, NF-κB↓, TNF-α↓, Iba-1↓, Bax↓, Bcl-2↑
Liu et al. (2015)	Ethidium Bromide induced demyelinating rat model of multiple sclerosis	GV6, GV9	EA, 4 mA, 2/100 Hz, 30 min/day	once a day, 7 days	MWM test: escape latency↓, platform crossing number↑	Demyelination tissue of spinal cord: NT-3↑, NG2/GFP double-positive cell↑, number of newborn myelin↑, normal myelin↑; MEPs: latency↓, amplitudes↑
Yang et al. (2010)	hSOD1G93A mice model of ALS	ST36	EA, 1 mA, 2 Hz, 30 min/day	Twice/week, 110 days	Rotarod test: time remaining on the rotating rod↑	Brain stem: NeuN-positive cell↑, Iba1 expression↓, MAP2 expression↑, active caspase-3, phospho-ERK↑, active-AKT expression↑; spinal cord: NeuN-positive cell↑, Iba1 expression-, Iba1-positive cell↑, MAP2 expression↑, TNF-α level↓, immunoreactive cell↓, phospho-p38 expression↓, phospho-ERK↑, active-AKT expression↑; facial nucleus of the brain stem: Iba1-positive cell↑, TNF-α level↓, immunoreactive cell↓

^#^*p* > 0.05; 6-OHDA, 6-hydroxydopamine; ACh, acetylcholine; ALS, amyotrophic lateral sclerosis; Akt, protein kinase B; APO, Apomorphine; Bax, Bcl-2-associated X; Bcl, B-cell lymphoma protein; BDNF, brain-derived neurotrophic factor; BOLD, Blood Oxygen Level Dependent; CAT, catalase; Cr, creatine; DA, dopamine; DAT, dopamine transporter; DNP, dinitrophenyl; EA, electricacupuncture; ENK, enkephalin; ERK, extracellular signal-regulated kinase; GABA, γ-aminobutyric acid; GAD67, glutamate decarboxylase 67; GCLM, glutamate-cysteine ligase modifier subunit; GDNF, glialcellline derived neurotrophic factor; GFAP, glial fibrillary acidic protein; GFRα-1, GDNF family receptor alpha-1; Glx, glutamate + glutamine; Glu, glutamine; GP, globus pallidus; GPe, global pallidus externa; GPi, globus pallidus interna; hPAP, ARE-driven reporter gene; HO-1, heme oxygenase-1; Iba-1, Ionized calcium binding adapter molecule 1; IL, interleukin; IOD, integral optical density; LTP, long-term potentiation; MA, manual acupuncture; MAP2, microtubule association protein-2; MDA, malondialdehyde; MEPs, Motor-Evoked Potentials; MFB, medial forebrain bundle; mGluR3, metabotropic glutamate receptor 3; MRI, Magnetic Resonance Imaging; MPTP, 1-methyl-4-phenyl-1,2,3,6-tetrahydropyridine; MWM, Morris water maze; NAA, N-acetylaspartate; NG2, nerve/glial antigen 2; NF-κB, nuclear factor kappa-B; NQO1, nicotinamide adenine dinucleotide phosphate quinone oxidoreductase; Nrf2, nuclear factor erythroid 2-related factor 2; NT-3, neurotrophin-3; OFT, Open Field Test; pAkt, phosphorylated Akt; pCREB, phosphorylated CREB; PD, Parkinson’s disease; Pitx3, Paired Like Homeodomain 3; PSD95, postsynaptic density protein 95; SN, substantia nigra; SNpc, substantia nigra pars compacta; SOD, superoxide dismutase; TH, Tyrosine hydroxylase; TNF-α, tumor necrosis factor-α; VGLUT-1, vesicular Glu transporter-1; VMAT2, vesicular monoamine transporter 2.

Animal research has reported that acupuncture alleviates behavioral disorders in PD model rats (Haiyang et al., 2023), enhances the number of TH-positive neurons in the SN, and decreases the deposition of Fe in the SN (Li et al., 2019). Regarding Tourette syndrome (TS), the “DA hypothesis” was proposed for the pathophysiology of TS (Nespoli et al., 2018), in which the hypersensitivity of DA receptors and the hyperactivity of dopaminergic neurons in the nigrostriatum were the basic mechanisms (Dwyer, 2018). In TS model mice, acupuncture reduced the overexpression of TH in the substantia nigra pars compacta (SNpc) and reduced behavioral stereotypies (Lin et al., 2019). Interestingly, there is a benign modulatory effect of DA after acupuncture treatment. In a PD mouse model, acupuncture enhanced dopamine release in the synaptic cleft in the basal ganglia neural circuit, which benefited postsynaptic dopamine neurotransmission (Kim et al., 2011). Moreover, the combination therapy of acupuncture and levodopa (L-DOPA) significantly improved movement speed and fine motor performance in Parkinsonian rhesus monkeys (Zhang et al., 2018). In addition, studies using the combination of acupuncture with a 50% reduced dose of L-DOPA (7.5 mg/kg) showed an equivalent effect with the standard dose of L-DOPA alone in motor function improvement, and the combination of acupuncture with L-DOPA was superior in behavioral benefits compared to L-DOPA alone (Kim et al., 2014). For basal ganglia-thalamocortical circuits, 6-hydroxydopamine (6-OHDA)-lesioned rats showed decreased D1R expression and upregulated D2R expression in the striatum. High-frequency (100 Hz) electroacupuncture (EA) inhibited the upregulation of D2R and significantly restored striatal D1R mRNA and protein levels (Rui et al., 2013). Considering the important role of D1R in the direct pathway and the dominant role of D2R in basal ganglia circuits, EA showed a benign effect in balancing dopaminergic neural circuits.

In the dopaminergic system, dopaminergic neuron loss in the SNpc is the major cause of PD. Sufficient studies have supported the crucial role of oxidative stress in the development of PD. Hence, reinforcing the antioxidative capacity of dopaminergic neurons is considered a practicable strategy for the prevention and treatment of PD. In 1-methyl-4-phenyl-1,2,3,6-tetrahydropyridine (MPTP)-induced PD model mice, lipid and protein oxidation was upregulated in the striatum, while antioxidants were downregulated. After applying high-frequency EA (100 Hz), there was a significant decrease in hydrogen peroxide and malondialdehyde (MDA) levels and an increase in glutathione, catalase (CAT) and superoxide dismutase (SOD) levels in the striatum. Meanwhile, EA enhanced the nuclear factor erythroid 2-related factor 2 (Nrf2)-regulated downstream antioxidative response and upregulated the expression of nicotinamide adenine dinucleotide phosphate quinone oxidoreductase (NQO1) and heme oxygenase-1 (HO-1) in the midbrain and/or striatum (Lv et al., 2015). These findings support the antioxidant role of acupuncture in the dopaminergic system.

Neurotrophic factors are important endogenous biological mediators due to their crucial roles in neural development and functional regulation. Targeting neurotrophic factors in neural circuits has been reported to facilitate recovery in neurodegenerative diseases. For instance, brain-derived neurotrophic factor (BDNF) and glial cell line-derived neurotrophic factor (GDNF) have been proven to benefit the survival and development of dopaminergic neurons and thus may bolster PD treatments through the nigrostriatal pathway. In PD model mice, a 3-week EA in GV20 and GV14 ameliorated motor impairments and dopaminergic neuron loss and increased the levels of BDNF and GDNF in the substantia nigra and striatum (Pak et al., 2019). Similarly, another study conducted EA in GB34 and LR3 in PD model mice showed consistent results in behavioral improvement and BDNF enhancement (Lin et al., 2017).

### 3.2 The impact of acupuncture on the glutamatergic system

In PD-related studies (Table 1; Figure 1), a 24-session EA treatment at GV14 and GV20 showed significant improvement in motor dysfunction. Meanwhile, EA upregulated the expression of NMDA receptor subunit 2B (NR2B) and reversed the decrease in dendritic arborization, spine density, and LTP in the striatum. In addition, striatal glutamate and vesicular Glu transporter-1 (VGLUT-1) were downregulated after EA intervention in corticostriatal glutamatergic projections (Li et al., 2022). Regarding the classical pathway of the basal ganglia circuit, studies have demonstrated the effect of acupuncture on PD by modulating the Glu system in the motor cortex-subthalamic nucleus (M1-STN) pathway. In 6-hydroxydopamine (6-OHDA)-lesioned rats, there is a significant increase in glutamate content in the lesion-side nigrostriatal system, and a decreased metabotropic glutamate receptor 2/3 (mGluR2/3) protein level and mGluR3 mRNA expression were observed. Four weeks of EA treatment on GV14 and GV20 alleviated motor deficits, downregulated glutamate levels in the cortex and striatum, and rescued mGluR3 mRNA expression and mGluR2/3 protein expression (Jia et al., 2017). Another study reported the glutamate-dependent mechanism focused on VGLUT-1. In a PD rat model, 100-Hz EA treatment significantly restored the loss of VGLUT-1 in the STN and alleviated motor symptoms induced by 6-OHDA (Wang et al., 2018). Apart from the direct influence of the Glu system on PD, acupuncture showed a protective effect on the dopaminergic system by modulating the Glu system (Li et al., 2022). Jia’s team proved EA at 100 HZ reduced the mRNA expression of glutamate decarboxylase 67 (GAD67) and enhanced the TH-positive neurons in SN in the rat model of PD (Jia et al., 2009).

These findings proved the direct regulation effect of acupuncture on the glutamate receptors, and the indirect modulation effect on the dopaminergic system via the glutamatergic system.

### 3.3 The impact of acupuncture on the GABAergic system

**Figure 4:**
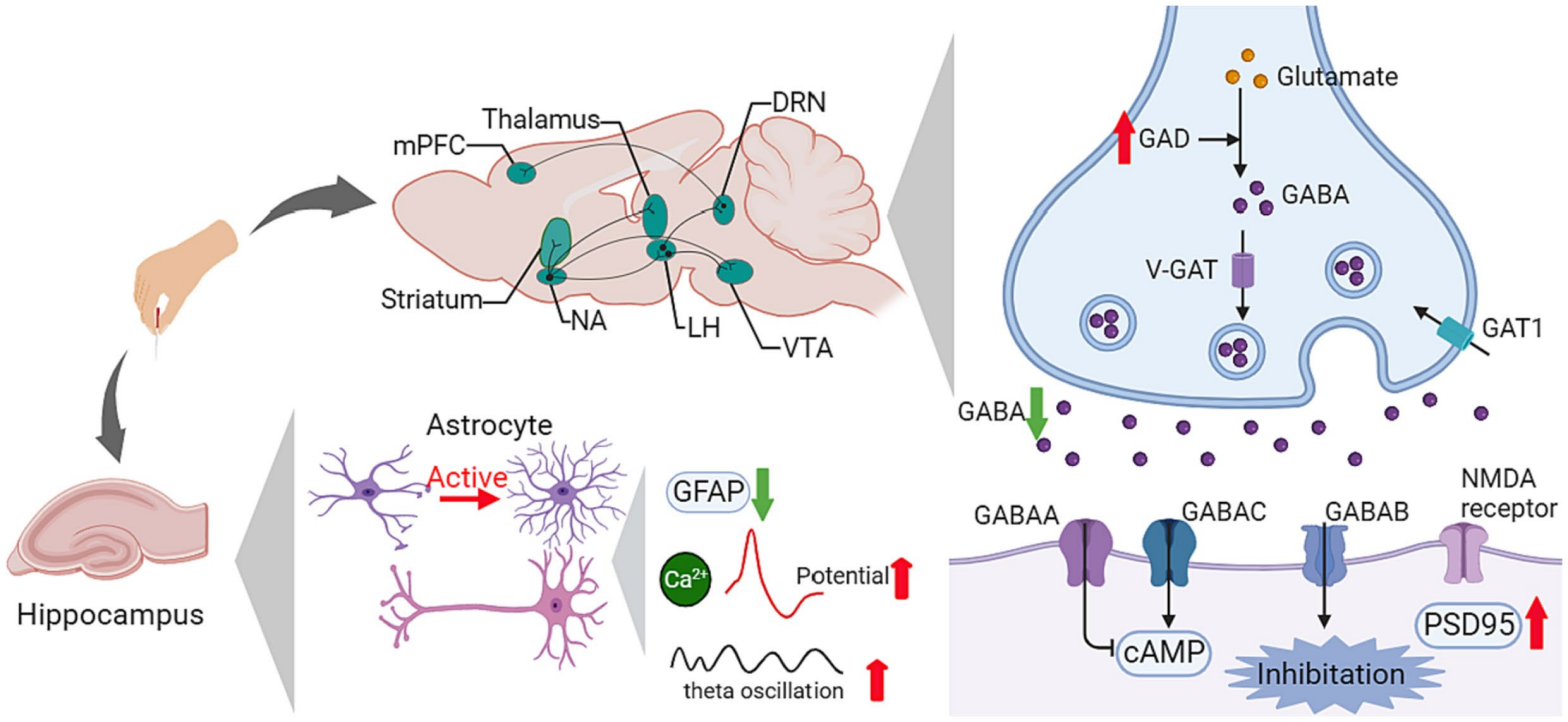
Influence of acupuncture on the GABAergic system. AMPA, α-amino-3-hydroxy-5-methyl-4-isoxazole propionic acid; cAMP, cyclic adenosine monophosphate; DRN, dorsal raphe nucleus; GABA, γ-aminobutyric acid; GAD, glutamate decarboxylase; GAT1, GABA transporter 1; GFAP, glial fibrillary acidic protein; LH, lateral hypothalamus; MAPK, mitogen-activated protein kinase; mPFC, medial prefrontal cortex; NA, nucleus accumbens; NMDA, N-methyl-D-aspartate; PSD95, postsynaptic density protein 95; V-GAT, Vesicular GABA transporter; VTA, ventral tegmental area.

Given that GABAergic neurons account for the impressive modulation of synapses, in the basal ganglia neural circuit, they are crucial transmitters of inhibitory projections in both the direct pathway and indirect pathway. The dysfunction of GABA has been revealed to be a vital process in PD pathogenesis. Recent studies demonstrated that acupuncture facilitated the normalization of the GABA content in PD (Table 1; Figure 4).

In 6-OHDA-induced PD model rats, elevated GABA content was found in the lesion-side cortex and striatum. After EA for 24 sessions, the GABA ratio of the normal side to the lesion side in the cortex was reduced significantly (Du et al., 2011). Another study investigated the therapeutic mechanism of EA in a rat hemiparkinsonian model and reported similar results. High-frequency (100 Hz) EA attenuated the medial forebrain bundle (MFB) lesion-induced increase in midbrain GABA content, increased GABAergic inhibition in the basal ganglia, and improved motor coordination (Jia et al., 2010). Regarding the mutual influence between the GABAergic system and the dopaminergic system in the PD process, PD treatment normalized the GABA content in the SNr. EA had a beneficial effect on these two systems. In Kim’s study, EA showed an equivalent effect on motor function improvement to L-DOPA, and the combination treatment of L-DOPA and EA ameliorated the abnormally increased GABA in the SNr (Kim et al., 2014).

### 3.4 The impact of acupuncture on the serotonergic system

In PD, the degeneration of suture nucleus neurons and the decrease in serotonergic innervation are mutual intervention processes, of which the dysfunction of 5-HTergic neurons affects the conversion of L-DOPA to DA, thus leading to the aggravation of PD (Nemade et al., 2021). In the rat PD model, Sun’s study reported that EA reduced the abnormally elevated acetylcholine (ACh) and Glu in the lesioned side of the striatum, and the Glu level was correlated with the survival ratios of dopaminergic neurons in the SNpc and behavioral improvement (Sun et al., 2012). In Ning’s study (Ning et al., 2023), EA alleviated motor and depressive symptoms. At the same time, EA reversed the decreased 5-HT and DA content in the midbrain of PD rats, attenuated the increased α-syn, activated the mTOR pathway, and inhibited autophagy in the striatum (Table 1; Figure 2).

### 3.5 The impact of acupuncture on the cholinergic system

In PD, cholinergic interneurons within the striatum play an important role in balancing dopamine signaling and regulating movement. In the striatum, cholinergic interneurons form axon-axonal connections with afferent glutamatergic and dopaminergic terminals, exerting influences on striatal function together with GABAergic interneurons and DA neurons (Liu, 2020). As the neurotransmitter, ACh has been recognized as a critical mediator in the transmission of striatal glutamate, which influences the release of DA in the striatum. Currently, sufficient findings have proven the contribution of the cholinergic system to the pathophysiology of PD. However, there is limited evidence in acupuncture-related studies, which requires further investigation (Table 1). In Sun’s research, high-frequency EA (100 Hz) stimulation at GV14 and GV20 ameliorated rotational behavior in Parkinsonian rats, attenuated the abnormally increased content of Glu and ACh in the lesioned side of the striatum, and enhanced the survival of dopaminergic neurons in the SNpc (Sun et al., 2012).

### 3.6 The impact of acupuncture via the microglia-related pathways

In PD-related studies (Table 1), Jang’s team proved that acupuncture at GB34 and ST36 improved motor function in PD model mice, restored dopaminergic fiber and neuron damage induced by MPTP, attenuated the overexpression of microglia and astrocytes, and ameliorated the inflammatory responses and apoptosis in the striatum and the substantia nigra (Jang et al., 2020b). In Lv’s study, EA at ST36 and SP6 demonstrated consistent results, relieving abnormal movement in MPTP mice and reducing microglial activation and astrogliosis in the striatum and midbrain (Lv et al., 2015). In addition, acupuncture demonstrated an adjunctive role in the transplantation therapy of multiple sclerosis using bone marrow mesenchymal stem cells. EA at GV9 and GV6 increased neurotrophin-3 (NT-3) levels, facilitated oligodendrocyte-like cell differentiation from grafted NT-3 and retinoic acid (RA) preinduced mesenchymal stem cells, and promoted remyelination in the demyelinated spinal cord. Meanwhile, EA speeds up the conduction of cortical motor-evoked potentials, improving nerve conduction function in multiple sclerosis (Liu et al., 2015). Regarding amyotrophic lateral sclerosis (ALS), EA at ST36 reduced microglial cell activation and restored motor neuronal cell survival in the brain stem and spinal cord of mutant ALS model mice (Yang et al., 2010).

The above studies provide evidence for acupuncture’s therapeutic mechanism of protecting dopaminergic neurons, interfering with inflammatory responses and apoptosis in PD, prompting remyelination and nerve conduction function in multiple sclerosis, and facilitating neuronal survival in ALS.

## 4 Neural circuit mechanism of acupuncture on cognitive impairment via different neuronal and glial systems

### 4.1 The impact of acupuncture on the glutamatergic system

In animal studies, acupuncture showed indeed effect on improving short-term memory, long-term memory, and spatial memory ability in mice models of cognition impairment. Beneath acupuncture’s effect, the potential mechanism might related to the regulation of Glu expression level (Table 2; Figure 1).

**Table 2:** The effect and mechanisms of acupuncture on cognitive disorders in neurodegenerative diseases.

Author	Animal model/participant	Acupoint	Acupuncture type	Acupuncture session	Behavioral test	Outcomes
Glutamatergic system						
He et al. (2022)	METH withdrawal mice model of spatial memory impairment	GV29, GV20	EA, 200 Hz with 1–2 mA, 30 min/day	14 days	MWM: time in probe test↓; time in OLM test↑	dCA1: extracellular Glu↓, c-Fos-positive neurons↓; C3-positive astrocytes↓, reactive astrocytes↓, C3 protein↓, GLT-1-positive astrocytes↑, GLT-1 protein↑; Schaffer projections from dCA3 to dCA1: number of c-Fos-positive presynaptic neurons^#^, VGLUT-1-positive staining^#^
Hou et al. (2022)	SAMP8 mice model of AD	GV20, ST36	EA,1 mA, 10 Hz, 30 min/day	14 days	Y Maze Test: correct spontaneous alternation rate↑; MWM test: movement paths↓, escape latency↓, percentage of time spent in target quadrant↑, number of platform crossings↑	CSF: OxA↓, Glu↓; hippocampus: Nissl bodies (CA1, CA3)↑; OxA protein (hippocampus, lateral hypothalamus)↓, cAMP↑, pPKA/PKA↑, pCREB/CREB↑, GluN1↑, GluN2A↑, GluA2↑, SYP↑, PSD95 ↑, number of synapse↑, synaptic density↑
Lin et al. (2018)	APP/PS1 transgenic mice model of AD	GV20	EA, 100 Hz, 1/20 mA, 30 min/day	6 days/week, 4 weeks	Escape latency↓, number of mice crossing the platform↑ Step-down avoidance test: latency↑, number of errors↓	Left hippocampus: NAA/Cr↑, Glu/Cr↑; hippocampus, CA1 and CA3 areas: surviving neurons↑; hippocampus: BDNF↑, TrkB↑
GABAergic system						
Li et al. (2023)	5xFAD mice model of AD	GV20, GV14	EA, 2 Hz, 1 mA, 30 min/day	5 days	MWM test: escape latency↓ NOR task: time↑	Hippocampus: gephyrin protein↑, synapsin 1^#^, PSD95^#^, GAD65↑, SST↑, PV^#^; dorsal CA1: CR↑ cell↑, sIPSCs frequency↑, sEPSCs frequency↑, sEPSCs area↑, sEPSCs amplitude^#^, charge ratio of sEPSC/sIPSC↓, theta oscillation↑, gamma oscillation↑; dorsal hippocampus: SST^+^ cell↑
Serotonergic system						
Xiao et al. (2023)	D-galactose induced rat model of AD	GV20, ST36	EA, 50 Hz, 1 mA, 20 min/day	once a day, 8 weeks	MWM test: escape latency↓, exploration time↑	colon: 5-HT levels↓, Trp↑ hippocampus: 5-HT levels↑, Trp↑, 5-HT6R↓, JNK↓, p-JUNK↓, c-JUN↓, p-c-Jun↓
Cholinergic system						
Li et al. (2022)	5xFAD mice model of AD	GV 20, GV 14	EA, 2/20 Hz, 1 mA, 30 min/day	5 days/week, 4 weeks	OFT^#^: traveled distance, average speed, time spent in the central part; NOR^#^: object exploration; Novel location recognition: location exploration↑; Location discrimination task: Must touch training trials^#^, PIT accuracy^#^, LDR trials↓	MS/VDB: NAA/Cr↑, Cho/Cr↑; AChE↓, VAChT↑, Aβ fraction ratio↓^#^ DG: NAA/Cr↑, Cho/Cr↑; DG, MOD of AChE^#^, MOD of VAChT^#^, ChAT↑, Aβ fraction ratio↓, M1^+^ cell↑ hippocampus: DCX^+^ cell↑, Neuro-D1^+^ cell↑
Lee et al. (2014)	Scopolamine induced rat model of memory impairment	GV20	MA	-	MWM test: escape latency↓	Hippocampus: ChAT↑, BDNF↑, CREB↑, CHT1↑, VAChT↑, BDNF mRNA↑, CREB mRNA↑
Lee et al. (2012)	Corticosterone induced rat model of spatial cognitive impairment	HT7/TE5	MA	-	MWM test: escape latency↓; OFT: line crossing number^#^	Hippocampus: cholinergic neurons↑, BDNF mRNA↑, CREB mRNA↑ hippocampal CA1 area: ChAT-immunopositive neuron↑, AchE-immunopositive fibers↑; hippocampal CA3 area: AchE-immunopositive fibers↑; Medial septum: ChAT-immunopositive neuron numbers↑
Glial cells						
Xie et al. (2021)	Aβ1-42 induced rat model of AD	GV20	EA, 10 mA, 20 Hz, 30 min/day	6 times/week, 3 weeks	MWM test: escape latency↓, swimming speed↑, swimming distance↓, probe times of trails↑	CA1: activation of astrocytes↓, Iba1^+^ iNOS^+^ cell↓, Iba1^+^ Arg1^+^ cell↑, GFAP^+^ IL-1β^+^ cell↓, GFAP^+^ TNF-α^+^ cell↓, GFAP^+^ IL-6 cell↓, Iba1^+^ IL-4^+^ cell↑, Iba1^+^ IL-10^+^ cell↑, GFAP^+^ IL-4^+^ cell↑, GFAP^+^ IL-10 cell↑, Iba1^+^ p65^+^ cell↓, GFAP^+^ p65^+^ cell↓, Iba1^+^ STAT6^+^ cell↑, STAT6 mRNA expression↑; DG: activation of astrocytes↓, Iba1^+^ iNOS^+^ cell↓, Iba1^+^ Arg1^+^ cell↑, IL-1β^-#^, TNF-α↓^#^, Iba1^+^ IL-6^+^ cell^#^, GFAP^+^ IL-1β^+^ cell↓, GFAP^+^ TNF-α^+^ cell↓, GFAP^+^ IL-6^+^ cell↓, Iba1^+^ IL-4^+^ cell↑, Iba1^+^ IL-10^+^ cell↑, GFAP^+^ IL-4^+^ cell↑, GFAP^+^ IL-10 cell↑, GFAP^+^ p65^+^ cell↓, Iba1^+^ STAT6^+^ cell↑, STAT6 mRNA expression↑
Li M. et al. (2020)	SAMP8 mice model of AD	GV20, GV29	EA, 2 Hz, 0.1 mA, 15 min/day	once a day, 15 days	MWM test: escape latency↓, platform crossing number↑	Hippocampus: TREM2 protein↑
Wang et al. (2020)	SAMP8 mice model of AD	GV29, LI20	EA, 15 Hz, 1.5 mA, 10 min/day	6 days/week, 4 weeks	MWM test: escape latency↓, path length of place navigation trial↓, swimming time↑	Hippocampus: Aβ protein↓, hyperphosphorylated tau proteins↓, P-tau/tau↓^#^, integrated density of Iba1^+^ cell↓, activated microglia area↓; CA1: SYP expression content↑
Cai et al. (2019)	5xFAD mice model of AD	KI3	EA, 1 mA, 2 Hz, 15 min/day	3 times/week, 2 weeks	NOR test: percentage of exploration time of the novel object↑; Y maze test: spontaneous alternations↑	Frontal cortex: mean glucose level↑; prefrontal cortex: CD11b↓, GFAP↓, COX2↓, HO-1↓, transferrin↓, Bax↓, LTP↑, synaptophysin, PSD-95↑, synaptic structures↑, APP↓, APOE↓^#^, BACE↓^#^, Aβ1–42 levels↓, CD68^+^ cell/Aβ plaque↓; cortex: mean glucose level↑; hippocampus: mean glucose level^#^, LTP↑; hypothalamus: mean glucose level↑
Tang et al. (2019)	Aβ1-42 induced rat model of AD	GV20, BL23	MA, 20 min/day	once a day, 8 weeks	MWM test: escape latency↓, platform crossing number↑	Hippocampal dentate gyrus: neuronal edema↓, astrocytes edema↓
Wang et al. (2014)	APP/PS1 transgenic mice model of AD	GV20	EA, 2/15 Hz, 1 mA, 30 min/day	5 days/week, 4 weeks	MWM test: escape latency↓, swimming speed^#^, time spent in the target quadrant↑	GFAP↓, NDRG2↓
Han et al. (2018)	LPS-induced mice model of cognitive impairment	GV20	EA, 2/100 Hz, 4 mA, 30 min/day	7 days	MWM test: escape latency↓, platform crossing number↑	Hippocampus: α7nAChR positive neurons↑, ACh content↑, ChAT↑, AChE activities↓, MDA↓, H_2_O_2_↓, CAT activity↑, GSH content↑, IL-1β↓, IL-6↓, TNF-α↓

^#^*p* > 0.05; 5 × FAD, five familial Alzheimer’s disease; α7nAChR, α7 nicotinic acetylcholine receptors; Aβ, amyloid-β; ACh, acetylcholine; AD, Alzheimer’s disease; APOE, apolipoprotein E; APP/PS1, amyloid precursor protein/presenili1; Arg1, M2 marker Arginase 1; BACE, β-site amyloid precursor protein cleaving enzyme 1; Bax, Bcl-2-associated X; BDNF, brain-derived neurotrophic factor; C3, compliment component 3; cAMP, cyclic adenosine monophosphate; CAT, catalase; CD11b, cluster of differentiation 11b; Cho, Choline; Cr, creatine; CREB, cAMP response element-binding protein; CSF, cerebrospinal fluid; COX2, cyclooxygenase 2; dCA1, dorsal CA1; EA, electricacupuncture; GABA, γ-aminobutyric acid; GFAP, glial fibrillary acidic protein; GLT-1, glutamate transporter 1; Glu, glutamine; GSH, glutathione; H_2_O_2_, hydrogen peroxide; IL, interleukin; iNOS, inducible nitric oxide synthase inos; LDT, Location Discrimination Task; LPS, lipopolysaccharide; LTP, long-term potentiation; MA, manual acupuncture; MDA, malondialdehyde; MEPs, Motor-Evoked Potentials; MFB, medial forebrain bundle; mGluR3, metabotropic glutamate receptor 3; MRI, Magnetic Resonance Imaging; MS/VDB-DG, medial septal and vertical limb of the diagonal band and dentate gyrus; mTOR, mammalian target of rapamycin; MPTP, 1-methyl-4-phenyl-1,2,3,6-tetrahydropyridine; MWM, Morris water maze; NAA, N-acetylaspartate; NG2, nerve/glial antigen 2; NDRG2, N-myc downstream-regulated gene 2; NeuN, neuron-specific nuclear protein; NLR, Novel Location Recognition; NOR, novel object recognition; OFT, Open Field Test; OLM, object location memory; ORT, Object Recognition Test; OxA, Orexin A; pCREB, phosphorylated CREB; PIT, punish incorrect training; PKA, cAMP response element-binding protein; PSD95, postsynaptic density protein 95; PV, parvalbumin; SAMP8, senescence-accelerated mice prone 8; sEPSCs, spontaneous excitatory postsynaptic currents; SST, somatostatin; STAT6, signal transducer and activator of transcription 6; STN, subthalamic nucleus; SYP, synaptophysin; TREM2, triggering receptors expressed on myeloid cells 2; Trp, tryptophan; TrkB, Tyrosine Kinase receptor B; VAChT, vesicular acetylcholine transporter; VGLUT-1, vesicular Glu transporter 1.

In the senescence-accelerated mouse prone 8 (SAMP8) model, a 14-session 10 Hz EA treatment restored the Glu level in the hippocampus, improved the synaptic structure, and enhanced synaptic transmission, thus ameliorating the memory deficit (Hou et al., 2022). In amyloid precursor protein/presenili1 (APP/PS1) mice, EA at GV20 demonstrated similar results. EA upregulated Glu metabolism and the survival rate of neurons in the hippocampus and alleviated cognitive dysfunction (Lin et al., 2018). In Methamphetamine (METH)-withdrawal mice, EA at GV20 and GV29 alleviated impaired spatial memory. Meanwhile, EA attenuated the increased Glu level in the dorsal CA1 (dCA1) of the hippocampus by enhancing astrocyte-mediated Glu clearance and upregulating the expression of glutamate transporter 1 and glutamine synthase, which are responsible for astrocytic glutamatergic transportation (He et al., 2022).

### 4.2 The impact of acupuncture on the GABAergic system

In AD, the impairment of GABA interneurons contributes to theE/I imbalance and related excitotoxicity, which accelerates amyloid plaque deposition and hyperphosphorylated tau sequestration, thus resulting in cognitive impairment. Meanwhile, based on electroencephalogram (EEG), the oscillatory activities in targeted brain regions are related to specific behaviors. In cognitive-related studies, the θ rhythm is regulated by working memory processes, and the γ rhythm is associated with cognitive processes (Hamm et al., 2015). Abnormal γ and θ rhythms in brain oscillatory activities have been demonstrated among patients with AD, while interfering with GABAergic interneurons could restore γ oscillation (Verret et al., 2012). In five familial Alzheimer’s disease (5 × FAD) mutation mice, EA at GV20 and GV14 ameliorates recognition learning and memory function deficits, restores glutamate decarboxylase 65 (GAD65) and somatostatin (SST) in the hippocampus, attenuates the loss of somatostatin-positive interneurons in the dorsal hippocampus, and reverses the decreased θ and γ brain oscillations (Li et al., 2023; Table 2; Figure 4).

### 4.3 The impact of acupuncture on the serotonergic system

In D-galactose-induced AD-like rats, an eight-week EA pretreatment of GV20 and ST36 showed a therapeutic effect on improving cognitive dysfunction. Meanwhile, there was a significant increase in hippocampal 5-HT levels and a decrease in 5-HT_6R_ expression after EA pretreatment. The upregulation of 5-HT_6R_ has been associated with cognitive dysfunction and participates in the c-jun N-terminal kinase (JNK) pathway to modulate downstream cell apoptosis. In Xiao’s study (Xiao et al., 2023), EA downregulated the abnormally increased expression of JNK-related molecules, including JNK, p-JNK, c-JUN, and p-c-Jun, in the hippocampus of AD rats (Table 2; Figure 2).

### 4.4 The impact of acupuncture on the cholinergic system

Acupuncture showed improvement in spatial recognition memory in cognition impaired model mice. As for the therapeutic mechanism, four main contributions have been reported on the impact of acupuncture on the cholinergic system: the neuronal protective effect on the basal forebrain-hippocampus circuit, benign regulation effect on the neural plasticity-related neurotransmitters and proteins, modulation of the inflammatory cholinergic-dependent pathway, and improvement of the oxidative stress activity.

**Figure 5:**
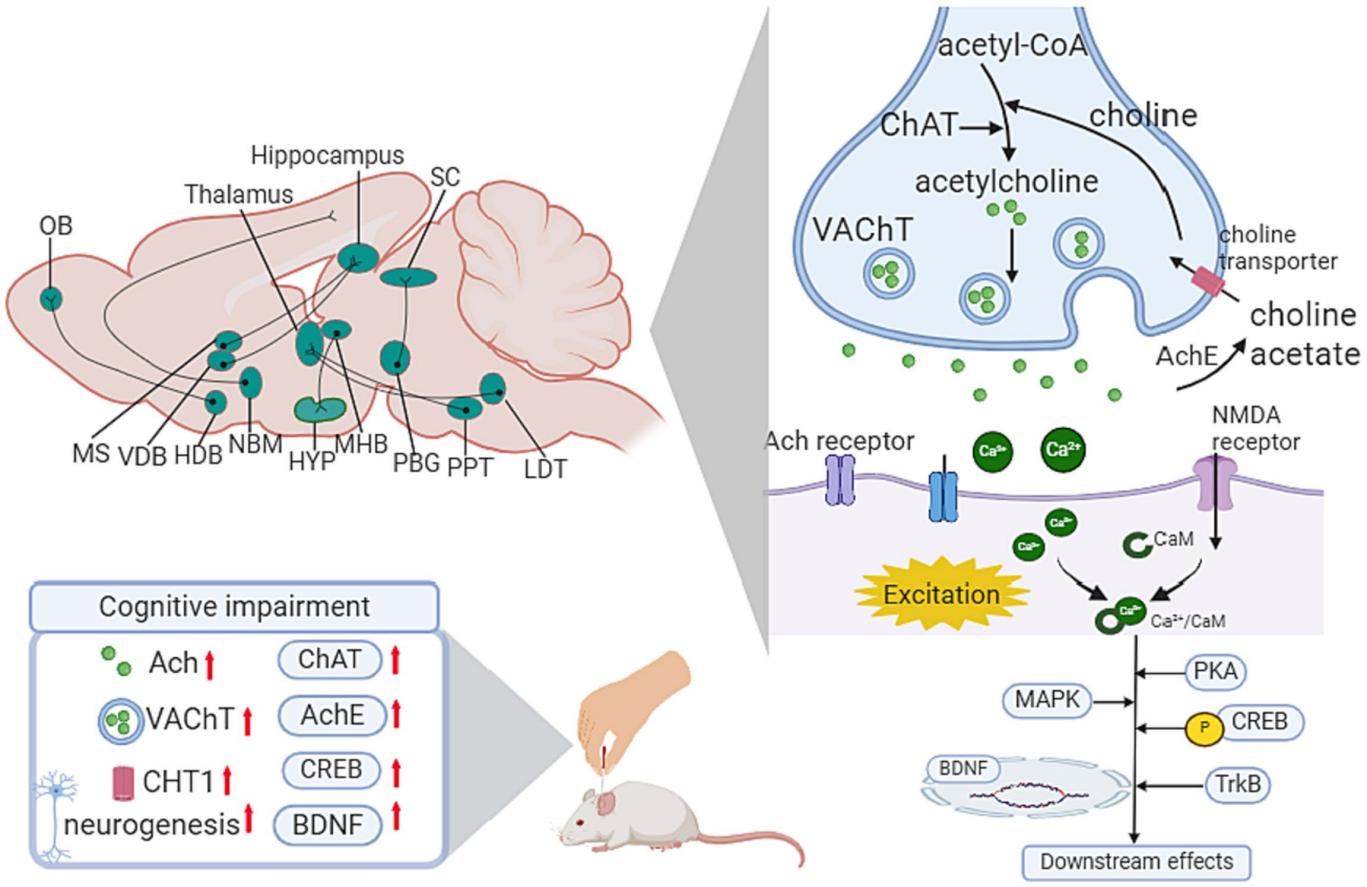
Influence of acupuncture on the cholinergic system. Ach, Acetylcholine; ChAT, choline acetyltransferase; AChE, acetylcholinesterase; BDNF, brain-derived neurotrophic factor; CaM, calmodulin; CHT1, choline transporter 1; CREB, cAMP response element-binding protein; HDB, horizontal diagonal band; HYP, hypothalamus; LDT, laterodorsal tegmental; MAPK, mitogen-activated protein kinase; MHB, medial habenula; MS, medial septal; NBM, Nucleus basalis of Meynert; OB, olfactory bulb; PBG, parabigeminal nucleus; PPT, peduculo-pontine tegmental nucleus; SC, superior colliculus; VDB, vertical limb of the diagonal band; VTA, Ventral tegmental area.

Focusing on the basal forebrain-hippocampus circuit, Li’s study (Li L. et al., 2021) highlighted the mechanism of EA treatment on the medial septal and vertical limbs of the diagonal band and dentate gyrus (medial septal/VDB-DG) cholinergic neural circuit. After applying EA treatment in 5 × FAD mice for 20 sessions, there was a significant improvement in spatial recognition memory and the medial septal/VDB-DG cholinergic neural circuit regulation, which promoted hippocampal neurogenesis in DG. Regarding mechanisms via synaptic plasticity pathways, one of Lee’s studies reported the cholinergic system-related mechanism of EA therapy in scopolamine-induced cognitive impairment rats. After conducting 14 sessions of EA on GV20, neuronal impairment and memory dysfunction improved significantly. Meanwhile, EA increased choline acetyltransferase (ChAT), BDNF and CREB protein levels and restored the decreased mRNA expression of choline transporter 1(CHT1), vesicular acetylcholine transporter (VAChT), BDNF and CREB in the hippocampus (Lee et al., 2014). Another study conducted by their team proved that EA at HT7 attenuated the loss of cholinergic neurons, decreased cholinergic immunoreactivity, and increased the mRNA expression of BDNF and CREB in a corticosterone-induced memory dysfunctional rat model (Lee et al., 2012). In addition, Liu’s study demonstrated the anti-inflammatory cholinergic-dependent pathway in the neuroprotection mechanism of acupuncture, thus improving cognitive function. In addition, accumulating research has focused on oxidative stress and neuroinflammation signaling pathways in EA-mediated prevention of cognitive impairment. Cao’s team observed neuronal damage, inflammation, and downregulation of α7nAChR in the hippocampus of chronic cerebral hypoperfusion (CCH) rats. EA restored neuronal survival and regulated the expression of α7nAChR, together with its downstream Janus Kinase 2 (JAK2)/Signal Transducer and Activator of Transcription 3 (STAT3) pathways. Meanwhile, EA suppressed microglia activation and inflammatory cytokines (Cao et al., 2021). In the lipopolysaccharide (LPS)-induced cognitive impairment mouse model, EA pretreatment on GV 20 ameliorated the decrease in α7nAChR protein, ACh content and ChAT activity and enhanced the activity of AChE in the cholinergic pathway of the hippocampus. Regarding oxidative stress, EA inhibited the expression of MDA and hydrogen peroxide (H_2_O_2_) and downregulated the levels of catalase (CAT) and glutathione (GSH) in the hippocampus (Table 2; Figure 5).

### 4.5 The impact of acupuncture via the astrocyte-related pathways

Acupuncture demonstrated improvements in cognitive impairment including delaying memory decline and enhancing memory capacity. Current studies have investigated the role of astrocytes in acupuncture’s effect mechanisms in AD, such as protecting astrocytes and neurons, and modulating the expression level of AD-related proteins (Table 2; Figure 4).

Tang’s team showed that acupuncture at GV20 and BL23 altered morphological damage in astrocytes and neurons in the hippocampal dentate gyrus and improved cognitive dysfunction in Aβ_1-42_-induced AD model rats (Tang et al., 2019). In Wang’s study, EA at GV20 for 4 weeks suppressed astrocytosis in APP mice, ameliorated AD-induced cognitive impairment and memory decline, and attenuated the upregulation of glial fibrillary acidic protein (GFAP) in reactive astrocytes, implying the therapeutic pathway of EA via astrocytic alteration (Wang et al., 2014). Similar results have been reported in Li’s study, which applied EA at ST36 in vascular dementia rats and found a decreasing tendency of astrocytes and an increased pyramidal neuron number in the hippocampal CA1 area (Li et al., 2015).

### 4.6 The impact of acupuncture via the microglia-related pathways

In cognitive impairment-related studies focusing on microglia, acupuncture elevated learning ability, memory capacity, and working memory. The potential mechanism related to the anti-inflammatory pathway, the modulation of the microglia activation, and the regulation of the AD-related protein expression (Table 2; Figure 1).

In an AD animal model, EA at GV20 and GV29 upregulated the expression of triggering receptors expressed on myeloid cells 2 (TREM2) in the hippocampus, ameliorating learning and memory dysfunction and protecting neurons (Li Y. et al., 2020). TREM2 contributes to neuroinflammation in AD (Kober and Brett, 2017) and is expressed by microglia in the brain (Wolfe et al., 2019). The TREM2-mediated anti-inflammatory pathway might be the potential mechanism by which EA exerts its neuroprotective effect. In AD-related studies, Xie’s team (Xie et al., 2021) summarized the effect of EA in inhibiting the activation of glia and the M2 phenotype polarization of microglia. Meanwhile, there was a reduction in pro-inflammatory cytokines and an elevation in anti-inflammatory cytokines after EA intervention in the hippocampus of AD rats. In addition, Cai’s study reported a co-occurrence reduction in microglia-mediated Aβ deposition and amyloid precursor protein in the prefrontal cortex of 5 × FAD rats after EA treatment (Cai et al., 2019). Similarly, Wang’s team (Wang et al., 2020) proved that the “olfactory three-needle” reduced the deposition of Aβ, upregulated the expression of synaptophysin (SYP), and inhibited the excessive activation of microglia in the hippocampus of SAMP8 mice. Regarding neuroinflammation, EA reduced the content of IL-1β, interleukin-6 (IL-6) and tumor necrosis factor-α (TNF-α) in the hippocampus (Han et al., 2018).

### 4.7 The impact of acupuncture via the OL-related pathways

Acupuncture showed a positive effect on the learning ability and memory function in cognitive impaired model animals, and the mechanism might be the enhancement of cerebral perfusion, the improvement of oligodendrocyte regeneration, and regulation of neurotrophin-4/5- tyrosine receptor kinase B (NT4/5-TrkB) signaling pathway.

In CCH rats, Zhang’s team observed learning and memory abilities and the number of Oli2-marked OL-positive cells after acupuncture of GV20 and EX-HN1 for 4 weeks. The study results indicated that acupuncture induced a significant increase in OL-positive cells in the corpus callosum, an improvement in the structure of cerebral white matter fibers, and decreased learning and memory dysfunction (Li Z. et al., 2021). Ahn’s study demonstrated similar results after applying EA at GV20 and GV14. The 7-session EA stimulation attenuated memory impairment, strengthened OL differentiation from OPCs, improved white matter structure recovery in the corpus callosum, and activated the NT4/5-TrkB signaling pathway (Ahn et al., 2016). Additionally, EA at GV20, GV16, and BL23 showed a benign effect on promoting the proliferation and differentiation of endogenous neural stem cells to neurons and oligodendrocytes in the hippocampal dentate gyrus of AD model rats (Table 2).

## 5 Acupuncture details

**Figure 6:**
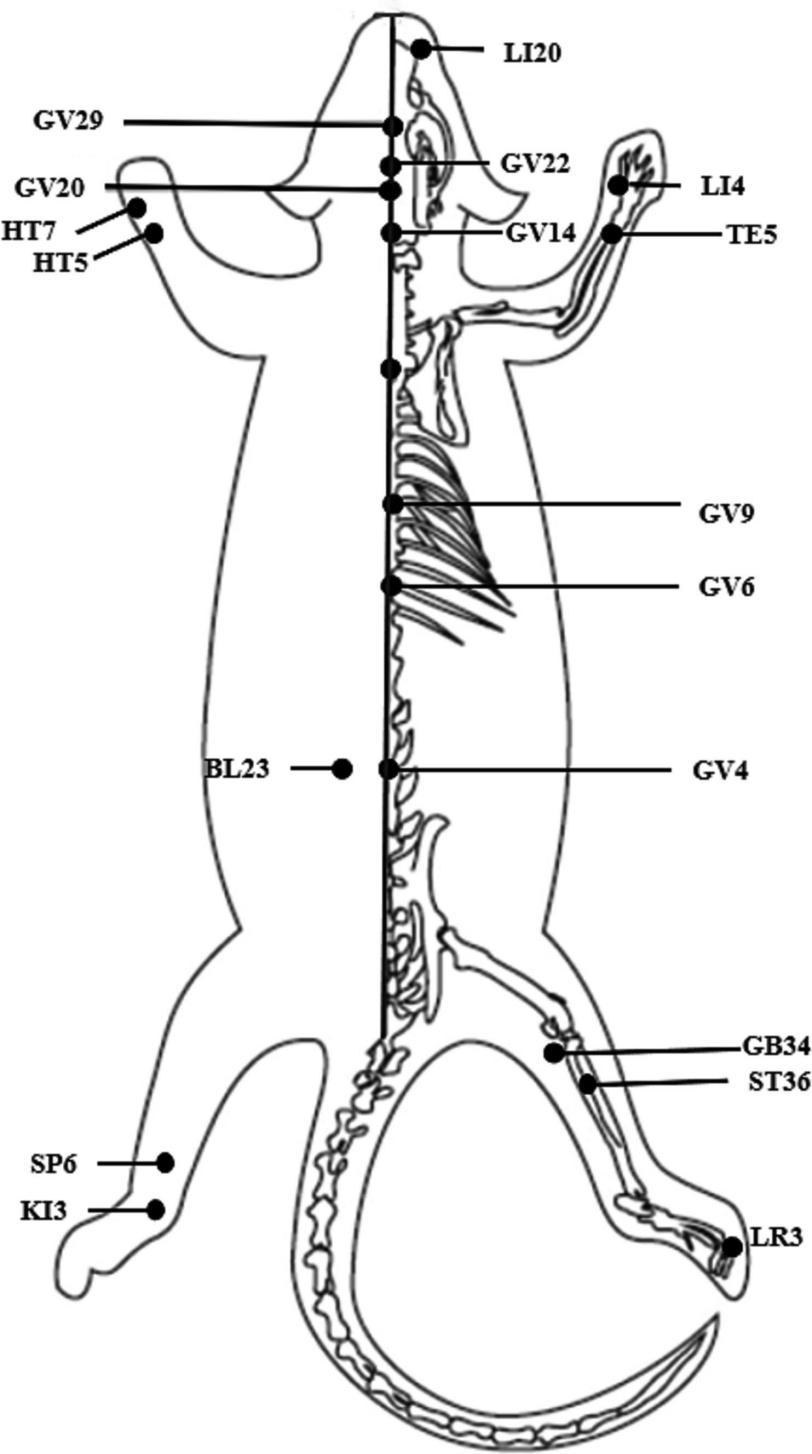
Acupoint position.

Among the included 34 studies, 7 studies used manual acupuncture, and the other 27 studies conducted electroacupuncture. The duration of acupuncture ranged from 7 days to 110 days, except for one study applying instant acupuncture. Twelve acupoints were mentioned in the movement disorders, of which the GV20 and GV14 were the most frequently used; and 10 acupoints were mentioned in the cognitive impairment, of which the GV20 and GV29 were the most frequently used. Figure 6 illustrates the position of the above acupoints.

## 6 Discussion

### 6.1 The characteristics and innovation of this study

This review summarized current evidence related to acupuncture’s therapeutic mechanism in neurodegenerative disease at the neural circuit level. We focused on movement disorder and cognitive impairment, which are the most frequently occurring symptoms in patients with neurodegenerative disorders. Regarding targeted disease, among the 38 included studies, 20 focused on motor disorders involving PD, ALS, and multiple sclerosis; 18 studies reported cognitive impairment, including AD, memory deficits, and spatial cognition impairment.

Previously, a literature review reported the therapeutic mechanism of acupuncture on PD in the dopaminergic neural pathway and summarized the efficacy and dopaminergic neuron protection of acupuncture (Zhao et al., 2021). In this review, we found that apart from the dopaminergic neural circuit, neural pathways such as the glutamatergic system, GABAergic system, and glial cells play roles in acupuncture improving motor disorders in PD.

In PD-related motor deficit studies, current findings support the hypothesis based on the basal ganglia circuit, and the targeted pathways projecting from the midbrain SNpc, striatum, cortex, and thalamus, of which the direct pathway comprises the basal ganglia circuit and the indirect pathway of basal ganglia-thalamus-cortex (Mcgregor and Nelson, 2019). At the circuit level, electroacupuncture treatment enhanced brain region activation in the striatum, primary motor cortex (M1), cingulate gyrus, and global pallidus externa (GPe) (Zhang et al., 2018). The results supported were consistent with the classical circuit model in the pathogenesis of PD. In molecular studies, dopaminergic neurons and DA receptors provide a structural basis at the circuit level. Acupuncture indicated a beneficial impact on restoring the survival of dopaminergic neurons and the metabolism of DA receptors, especially in the protein level and mRNA expression of D1R and D2R, which are the functional receptors in the direct and indirect pathways. Regarding the input pathway of prefrontal cortical-striatum projections, acupuncture demonstrated a similar protective effect on dopaminergic neurons and facilitated the elimination of α-syn in the striatum.

Apart from the direct influence on the dopaminergic system, neurons with other neurotransmitters displayed indirect effects on acupuncture’s mechanistic pathways. In the PD process, current studies proposed the hypotheses that the overexcitation of glutamatergic neurons in cortex-striatum projections deteriorates the degeneration of dopaminergic neurons, and the impairment of Glu receptors inhibits the information transmission efficacy in synaptic communication. Acupuncture showed a benign modulation of LTP and synaptic plasticity in the cortex-striatum circuits. GABAergic neurons account for inhibitory information transmission as interneurons in the indirect pathway. The imbalance of E/I information conduction resulted in dysfunction of the motor control system. Acupuncture demonstrated benign regulation of GABAergic neurons in the output pathway of the basal ganglia, and the cerebellum-basal ganglia-cortical circuit.

For cognitive impairment, which is a frequently reported symptom caused by a variety of diseases with different phases, our review concentrated on neurodegenerative diseases, which share common pathogenesis and rehabilitation mechanisms to some degree. Previous review reported the mechanistic pathways of different interventions on acupoints, and summarized the molecular mechanisms of neuroprotective effects on cognitive deficits (Zhang Z. et al., 2023). This study focused on acupuncture and electroacupuncture details, and highlighted the frequently studied acupoints and their targeted mechanistic pathways.

For cognitive impairment, some hypotheses stimulating acupoints in periphery could change the neurochemistry/neurocircuitry in the brain of rodents. The hippocampal Schaffer projection and hippocampal DG were the most frequently reported circuits. At the circuit level, acupuncture attenuated memory deficits by modulating the E/I balance of hippocampal GABAergic interneurons and facilitated the modulation of brain oscillations. For the basal forebrain-hippocampus cholinergic neural circuit, acupuncture displayed benign regulation of the molecular metabolism of the medial septal/VDB-DG pathway, which contributed to pattern separation impairment in cognitive impairment (Li et al., 2022). There are bidirectional connections in the basal forebrain-hippocampus projection, of which the hippocampal interneurons receive input from GABAergic neurons and cholinergic neurons and project to the medial septal/VDB in the basal forebrain from the CA1 region (Zheng et al., 2023). In our review, we found that the medial septal/VDB-DG pathway was a modulation target of acupuncture via the cholinergic neural circuit. Interestingly, according to brain-network research, abnormal functional connectivity within the medial septal/VDB circuit has been reported in PD patients with mild cognitive impairment (MCI) (Zhang P. et al., 2023). In addition, the volumes of cholinergic cells in the basal forebrain showed a relationship with disorders of brain rhythm (α-rhythm activity, θ oscillations) in MCI (Rea et al., 2021), and AD (Liu et al., 2022).

At the molecular level, the imbalance of Glu metabolism prevented neural information transmission efficacy, and acupuncture showed a bidirectional benign regulatory effect on synaptic plasticity. On the one hand, in the METH withdrawal model of spatial memory impairment mice, acupuncture accelerated the elimination of abnormally enhanced extracellular Glu in the dCA1 and suppressed irregular spontaneous excitatory postsynaptic currents (EPSCs) in Schaffer projections. On the other hand, in AD model mice, acupuncture restored the level of Glu receptors and promoted synaptic density in the hippocampus. In addition, acupuncture regulated the brain-gut axis via the serotonergic system, improving the learning and memory ability of AD model rats (Xiao et al., 2023).

Regarding glial cells, there is limited evidence of their direct influences on neural circuits in specific neural circuits. The indirect effect on acupuncture-related mechanisms involved neuron protective effects and energy metabolic modulation through astrocytes and microglia. Recent studies have proposed a new communication pathway in the neuron–glia–neuron system, the energy signaling pathway. Apart from classical signaling molecules, energy support plays a vital role in astrocyte connections with neurons (Illes et al., 2019). In general, adenosine triphosphate (ATP) derived from astrocytes inhibits neuronal excitability and signal transmission by modulating synapse formation and energy demands (Lezmy, 2023). At the circuit level, astrocyte-derived ATP indicated an increase in LTP and LTD in Schaffer collaterals (Obot et al., 2023). In the VD model, acupuncture has been proven to restore the ATP level and attenuate mitochondrial dysfunction from the accumulation of Aβ (Su et al., 2018). However, few studies have reported the astrocyte-induced energy metabolic mechanism of acupuncture intervention. Microglia are the crucial target of acupuncture intervention in the neuroimmune system, mainly involving inflammation and oxidative stress. Current evidence supports the neuroprotective effect of acupuncture by mediating the activation and polarization of microglia. Nevertheless, there have been limited findings focusing on specific neural circuits, except for the Schaffer collaterals and DG in cognitive impairment-related studies and the nigra-striatum network in movement disorder studies. Given the contribution of glial cells to the development and repair of neural function, relevant explorations in this field are needed in further studies.

## 7 Limitation

There are some limitations in this review. Firstly, this literature review describes the neural circuit mechanism in different chemical transmitters under acupuncture therapy. Due to the study type, we reported the included studies in a qualitative way rather than a quantitative evaluation. Secondly, though motor disorder and cognitive impairment were the common symptoms in neurodegenerative disorders, acupuncture-related studies mainly focused on PD and AD, except for three studies on TS, ALS, and multiple sclerosis separately. Given the increasing number of patients with neurodegenerative diseases, further studies should pay more attention to these diseases. Thirdly, since the neural circuit-related studies were mainly conducted on animal models, our findings mainly focused on rodents-related research, evidence based on humans are needed in the future.

## 8 Conclusion

In this review study, we summarized the acupuncture-related neural circuits in different chemical transmitters and glial cells on motor disorders and cognitive impairment in neurodegenerative diseases. In movement dysfunction, dopaminergic circuits, such as the cortex-basal ganglia-midbrain circuit, were the direct target of acupuncture’s mechanistic pathway, which was interfered with by other chemical transmitters and glial systems, and the glutamatergic pathway played crucial roles at the synaptic level. For cognitive impairment, acupuncture showed a neuroprotective role via the glutamatergic and cholinergic systems at the circuit level, including the forebrain-hippocampus and hippocampal circuits, and facilitated glial cells. Given the high occurrence of motor disorder and cognitive impairment in neurodegenerative disorders, there are shared pathogenesis in these two symptoms, and the behavioral tests indicated coincident improvement after acupuncture intervention. Currently, there is limited evidence in neural circuit research on both motor disorders and cognitive impairment, and acupuncture’s mechanism at the circuit level is in the preliminary stage. Considering that the high coincidence of acupoint selection implied a common recovery process in neurodegenerative diseases, the common mechanistic pathways under acupuncture intervention should be explored.

## Author contributions

BL: Conceptualization, Writing – original draft, Formal analysis, Funding acquisition, Investigation, Methodology, Resources, Software. SD: Data curation, Validation, Writing – review & editing. HJ: Validation, Writing – review & editing. WZ: Validation, Writing – review & editing. BZ: Visualization, Writing – review & editing. YD: Project administration, Writing – review & editing. ZM: Funding acquisition, Project administration, Supervision, Writing – review & editing.

## Glossary

5 × FAD: Five familial Alzheimer’s disease
5-HT: 5-hydroxytryptamine
6-OHDA: 6-hydroxydopamine
ACh: Acetylcholine
AD: Alzheimer’s disease
Akt: Protein kinase B
ALS: Amyotrophic lateral sclerosis
AMPA: Aminomethylphosphonic acid
APP: Amyloid precursor protein
ATP: Adenosine triphosphateadenosine triphosphate
BDNF: Brain-derived neurotrophic factor
C3: Compliment component 3
CAT: Catalase
CCH: Chronic cerebral hypoperfusion
ChAT: Choline acetyltransferase
Cho: Choline
CHT1: Choline transporter 1
CNS: Central neural system
CREB: cAMP response element-binding protein
DA: Dopamine
dCA1: Dorsal CA1
DG: Dentate gyrus
EA: Electroacupuncture
EEG: Electroencephalogram
E/I: Excitatory and inhibitory
EPSCs: Excitatory postsynaptic currents
EPSP: Excitatory postsynaptic potential
ERK: Extracellular signal-regulated kinase
GABA: γ-aminobutyric acid
GABAergic: γ-amino butyric acid-ergic
GAD65: Glutamate decarboxylase 65
GAD67: Glutamate decarboxylase 67
GDNF: Glialcellline derived neurotrophic factor
GFAP: Glial fibrillary acidic protein
Glu: Glutamic acid
GPe: Global pallidus externa
GSH: Glutathione
H2O2: Hydrogen peroxide
HO-1: Heme oxygenase-1
IL: Interleukin
iJAK2: Janus Kinase 2
JNK: c-jun N-terminal kinase
L-DOPA: Levodopa
LPS: Lipopolysaccharide
LTD: Long-term depression
LTP: Long-term potentiation
L-LTP: Late long-term potentiation
MA: Manual acupuncture
MAPK: Mitogen-activated protein kinase
MCI: Mild cognitive impairment
MDA: Malondialdehyde
MFB: Medial forebrain bundle
mGluR3: Metabotropic glutamate receptor 3
mTOR: Mammalian target of rapamycin
MPTP: 1-methyl-4-phenyl-1,2,3,6-tetrahydropyridine
NMDA: N-methyl-D-aspartate
NQO1: Nicotinamide adenine dinucleotide phosphate quinone oxidoreductase
Nrf2: Nuclear factor erythroid 2-related factor 2
NT-3: Neurotrophin-3
NT-4/5: Neurotrophin-4/5
OL: Oligodendrocyte
Olig2: Oligodendrocyte Transcription Factor 2
OPCs: Oligodendrocyte progenitor cells
RA: Retinoic acid
PD: Parkinson’s disease
PI3K: Phosphatidylinositol 3-kinase
PKA: cAMP response element-binding protein
PS1: Presenili1
SAMP8: Senescence-accelerated mice prone 8
sEPSCs: Spontaneous excitatory postsynaptic currents
SN: Substantia nigra
SNpc: Substantia nigra pars compacta
SOD: Superoxide dismutase
SST: Somatostatin
STAT3: Signal Transducer and Activator of Transcription 3
STN: Subthalamic nucleus
SYP: Synaptophysin
TH: Tyrosine hydroxylase
TNF-α: Tumor necrosis factor-α
TREM2: Triggering receptors expressed on myeloid cells 2
TS: Tourette syndrome
VAChT: Vesicular acetylcholine transporter
VGLUT-1: Vesicular Glu transporter-1
TrkB: Tyrosine receptor kinase B
VDB: Vertical limb of the diagonal band.
